# Detouring the Undesired Route of *Helicobacter pylori*-Induced Gastric Carcinogenesis

**DOI:** 10.3390/cancers3033018

**Published:** 2011-07-25

**Authors:** Eun-Hee Kim, Kyung-Sook Hong, Hua Hong, Ki Baik Hahm

**Affiliations:** 1 Lab of Translational Medicine, Lee Gil Ya Cancer and Diabetes Institute, Gachon University of Medicine and Science, 7-45 Songdo-dong, Yeonsu-gu, Incheon 406-840, Korea; E-Mails: kimeh@gachon.ac.kr (E.-H.K.); sukihong@gachon.ac.kr (K.-S.H.); ruihongh@gmail.com (H.H.); 2 Department of Gastroenterology, Gachon Graduate School of Medicine, Gil Hospital, Incheon 406-840, Korea

**Keywords:** gastric cancer, inflammation, *Helicobacter pylori*, cancer prevention

## Abstract

Epidemiological and experimental evidence has emerged that a dysregulated inflammation is associated with most of the tumors, and many studies have begun to unravel the molecular pathways linking inflammation and cancer. As a typical example linking these associations, *Helicobacter pylori* (*H. pylori*) infection-associated atrophic gastritis has been recognized as precursor lesion of gastric cancer. The identification of transcription factors such as NF-κB and STAT3, and their gene products such as IL-8, COX-2, iNOS, cytokines, chemokines and their receptors, *etc* have laid the molecular foundation for our understanding of the decisive role of inflammation in carcinogenesis. In addition to the role as the initiator of cancer, inflammation contributes to survival and proliferation of malignant cells, tumor angiogenesis, and even metastasis. In this review, the fundamental mechanisms of *H. pylori*-induced carcinogenesis as well as the possibility of cancer prevention through suppressing *H. pylori*-induced inflammation are introduced. We infer that targeting inflammatory pathways have a potential role to detour the unpleasant journey to *H. pylori*-associated gastric carcinogenesis.

## 1. Introduction

Gastric cancer is a major contributor to cancer-related death worldwide. Although gastric cancer incidence and mortality have declined in most areas of the World, gastric cancer is still the fourth most common cancer worldwide and ranks the third most common cause of cancer-related deaths [1]. Among the etiologies of gastric cancer, the Gram-negative bacterium *Helicobacter pylori* (*H. pylori*) is a well-established etiologic factor and has been classified as a class 1 carcinogen because of its causative role in the development of gastric cancer [2]. Though the chronic infection by *H. pylori* generates a state of inflammation, majority of the subjects remain asymptomatic through their life [3]. Nonetheless, in a subset of the *H. pylori*-infected population the gastric inflammation may evolve toward chronic active gastritis, and be implicated in more severe gastric diseases such as chronic atrophic gastritis and intestinal metaplasia, known as a precursor of gastric carcinogenesis, peptic ulcers, mucosa-associated lymphoid tissue lymphoma, and gastric cancer. Irrespective of outcomes, the shared features are the ability of *H. pylori* to infect and live persistently in the human stomach eliciting a chronic inflammatory response, which may contribute to a role in determining the varied clinical outcomes of infection. In spite of the report that prophylactic eradication of *H. pylori* after endoscopic resection of early gastric cancer should be used to prevent the development of metachronous gastric carcinoma [4] and debates still exist, in general case of gastric cancer, the simple removal of the *H. pylori* etiological factor did not contribute to cancer prevention, but can attenuate the emergence of precancerous lesion. Therefore, still more information regarding the link between *H. pylori* infection and gastric cancer is required for advancement of our knowledge in this field. In this review, we focus on the host inflammatory response to this infection, with special emphasis on the chemoprevention by targeting the inflammation-induced carcinogenesis.

## 2. Gastric Inflammation after *H. pylori* Infection

There are two main mechanisms by which *H. pylori* (or its products) may produce gastric inflammation. Firstly, the organism may interact with surface epithelial cells, producing either direct cell damage or the liberation of epithelial-derived pro-inflammatory mediators. Secondly, *H. pylori*-derived products may gain access to the underlying mucosa through type IV secretion system, thereby directly stimulating host non-specific and specific immune responses involving the liberation of a variety of cytokine messengers [5]. In addition, *H. pylori* directly damage the surface epithelial layer, thereby contributing to changes in mucosal permeability and enhanced antigen exposure. Adherence of the organism to gastric epithelial cells has been known to be accompanied by loss of microvilli, irregularity of the luminal border, and intracellular changes including loss of cytoplasm, edema and vacuolation. In addition, surface epithelial degeneration correlates with the numbers of *H. pylori* in close contact with the epithelial plasma membrane, a finding that supports a direct toxic effect of bacterial products on epithelial cells [6]. However, *H. pylori*-specific direct damage is not enough to explain the inflammatory responses, as most persons colonized with disease-associated strains remain asymptomatic. Therefore, how much the host defense mechanism to microbial attack does operate may be particularly important in the early stages of *H. pylori*-induced inflammation.

**Figure 1.**
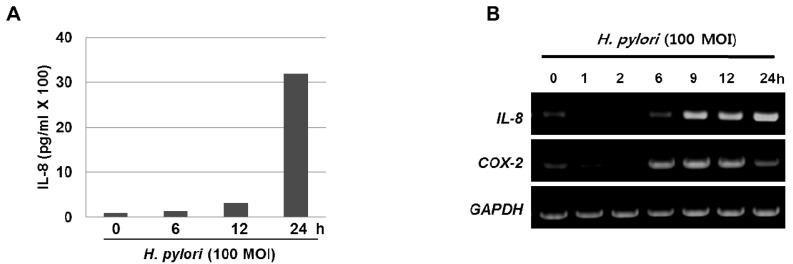
*Helicobacter pylori* induce the inflammatory signals *in vitro*. (**A**) The amounts of IL-8 were analyzed by ELISA assay in media supernatant from the AGS cells after treatment with *H. pylori* 100 MOI (multiplicity of infection) for 0, 6, 12, 24 h. (**B**) *H. pylori* induced the mRNA expressions of *IL-8* and *COX-2* in AGS cells. AGS cells were treated with 100 MOI *H. pylori* for the indicated time periods.

The inflammatory response-related host genes that have been most frequently studied in relation to gastric cancer are interleukin (*IL*) genes and tumor necrosis factor-alpha (*TNFA*). These cytokines are important mediators in gastric physiology and pathophysiology and could play important roles in the etiology of gastric cancer (*e.g.*, IL-1 controls stomach acidity, IL-8 stimulates the proliferation of endothelial cells, IL-10 down-regulates cytotoxic responses, and the pro-inflammatory cytokine TNF-α mediates inflammatory responses) [7]. *H. pylori* infection is also associated with the local production of chemokines and cytokines of which IL-6 is over-expressed at the margin of gastric ulcer in *H. pylori*-positive gastritis and is a cytokine that is central to the inflammatory arm of the innate immune response [8]. Moreover, our results clearly show that treatment with *H. pylori* induced the levels of IL-8 in the media supernatant as well as the mRNA expression in the gastric cancer AGS cells (Figure 1A and 1B). Host genetic factors also increase susceptibility to *H. pylori*-associated diseases. For instance, cytokine polymorphisms of the IL-1β and TNF-α genes are associated with increased susceptibility to peptic ulcer disease and gastric cancer [9,10]. In recently published meta-analyses, the authors showed increased cancer risk for IL-1RN2 carriers; the increased risk was specific to non-Asian populations and was seen for intestinal and diffuse cancers, distal cancers, and, to a lesser extent, cardiac cancers [7]. The IL-1RN22 genotype has been reported to cause high circulating IL-1 receptor antagonist (IL-1ra) and IL-1β levels, possibly resulting in a severe and prolonged inflammatory response. In another meta-analyses [11,12], investigators reported an increased risk for gastric cancer in carriers of *TNFA* among Caucasians, while increased risks for distal and intestinal cancers were also found in another meta-analysis [13], though with no further stratification by population. These reports indicate that interactions between specific host and microbial characteristics are biologically significant for the development of gastric cancer. On the basis of these case-control studies, it is apparent that *H. pylori* organisms are able to send and receive signals from their hosts, allowing host and bacteria to become linked in a dynamic equilibrium.

**Figure 2.**
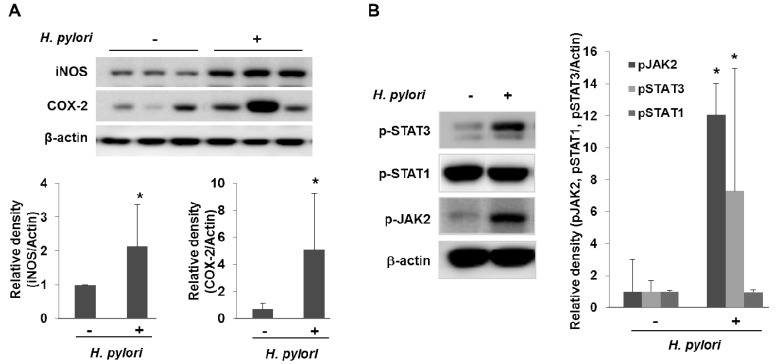
*Helicobacter pylori* induce the inflammatory signals *in vivo*. (**A**) Chronic infection of *H. pylori* induces the expression of COX-2 and iNOS in gastric mucosa *in vivo*. 5-week-old *IL-10*^-/-^ mice inoculated *H. pylori* and sacrificed after 28 weeks. The protein levels of COX-2 and iNOS were determined by Western blot analysis and quantification of COX-2 or iNOS immunoblot was normalized to that of β-actin followed by statistical analysis of relative image density. Representative 3 cases of 17 mice per group were presented. * *p* < 0.05. (**B**) Chronic infection of *H. pylori* induces the phosphorylation of JAK2 and STAT3 in gastric mucosa from *IL-10*^-/-^ mice. Western blots were probed with anti-phospho-STAT3 (p-STAT3), anti-p-STAT1 and anti-p-JAK2 antibodies. The most representative bands per group were presented. * *p* < 0.05.

In addition to stimulating cytokine production, *H. pylori* also activate pro-inflammatory enzymes such as cyclooxygenases (COX) and inducible nitric oxide synthase (iNOS). COX catalyze key steps in the conversion of arachidonic acid to endoperoxide (PGH_2_), a substrate for a variety of prostaglandin synthases that catalyze the formation of prostaglandins and other eicosanoids [14]. COX-1 is expressed constitutively in many cells and tissues, while COX-2 expression is inducible and can be stimulated by a variety of growth factors and pro-inflammatory cytokines, such as TNF-α, IFN-γ, and IL-1 [15]. COX-2 could contribute to carcinogenesis by promoting cell proliferation and angiogenesis as well as by protecting cells from apoptosis. Multiple lines of compelling evidences support that COX-2 is implicated in multi-stage carcinogenesis [15]. COX-2 expression is increased in gastric epithelial cells treated with *H. pylori*, within infected human gastric mucosa and further within gastric premalignant and malignant lesions, and COX inhibitors such as aspirin and other NSAIDs decrease the risk of distal gastric cancer [16]. *H. pylori* also activate iNOS, an enzyme that catalyze the production of nitric oxide (NO) from L-arginine. NO is an important cellular signaling molecule, having a vital role in many biological processes. Current information suggests that NO may contribute to carcinogenesis representing an important link between chronic inflammation and gastric carcinogenesis [17]. The postulated role of NO in carcinogenesis involves modulation of transcription factors (NF-κB, AP-1 pathways etc), direct oxidative DNA damage, stimulation of angiogenesis, inhibition of DNA repair enzymes, dysregulation of apoptosis and activation of oncogene expression [18]. We found recently a significant elevation of COX-2 and iNOS expression in the gastric tissues taken from *H. pylori*-infected IL-10 knockout mice as compared to non-infected gastric mucosa (Figure 2A).

Recent studies suggest that the IL-6/gp130/STAT3 (interleukin-6/glycoprotein 130/signal transducer and activation of transcription 3) pathway plays a role in the development of gastric cancer [19,20]. IL-6 exerts its biological activities through the receptor subunit gp130 which has functional modules to encompass four membrane distal Tyr(P) binding sites for the Src homology 2 domain of the latent transcription factors, STAT1 and STAT3 [21,22]. Lee and colleagues showed that *H. pylori* have preferential activation of the JAK/STAT3 pathway, via the IL-6 receptor gp130 after which activated JAK recruits STAT3 to the cell membrane gp130 complex, which then signals the nucleus via the STAT pathway. Translocated STAT3 to the nucleus induces *c-myc* expression and facilitates cell migration [23]. In accordance to this result, we also found a significant elevation of the phosphorylation of JAK2/STAT3 in the gastric mucosa from the *H. pylori*-infected IL10-knockout mice for 28 weeks (Figure 2B).

## 3. Link between *H. pylori*-Associated Inflammation and Gastric Carcinogenesis

Chronic inflammation is a well-known risk factor for cancer development. Since the discovery of the presence of leukocytes in neoplastic tissues by Rudolf Virchow in 1863, inflammation and cancer have been considered to be closely associated. The accuracy of Virchow's early observation is now more evident from multiple lines of studies suggesting the potential involvement of an inflammatory microenvironment in malignant transformation. Inflammatory cells and cytokines act as a tumor promoter that affects cell survival, proliferation, invasion, angiogenesis and chemo-resistance [24-26]. Approximately, 15% of all cancers are somehow linked to chronic inflammation-related process [27,28]. An earlier study based upon a transgenic mouse model demonstrated that elevation in the level of a single pro-inflammatory cytokine, IL-1β, is enough to induce gastric dysplasia or carcinogenesis, through the pathways of IL-1β and NF-κB. In this model, IL-1β activates myeloid derived suppressor cells in the stomach, whose mobilization and recruitment correlate with increased levels of IL-6 and TNF-α in serum and the stomach. Furthermore, the myeloid-derived suppressor cells also inhibit T-and B-cell proliferation. This provides a direct link between IL-1β, the myeloid-derived suppressor cells, and carcinogenesis via stepwise spontaneous inflammation, metaplasia, and dysplasia [29].

Although there has been a tendency towards a decrease in gastric cancer for the past 80 years, it is still the second highest cause of death following lung cancer. In Korea, about 25-30 people out of every 100,000 develop gastric cancer regardless of their sex, and its mortality rate is ranked as the highest. However, the discovery of a causative pathogen of gastric cancer, *H. pylori*, has raised hopes for preventing gastric cancer through eradication or regulation of the bacterium. Although *H. pylori* is a class I carcinogen, gastric cancer is not prevented by *H. pylori* eradication in all patients. This can be explained by the relationship between gastritis, metaplasia and gastric cancer, whereby prevention of *H. pylori*-associated carcinogenesis only benefits those in whom the malignant process has not begun [30]. That is, even though *H. pylori* is completely eradicated, gastric inflammation remains, thus it seems that amelioration of gastric inflammation itself seems to be far more essential in achieving cancer prevention than eradication of the pathogen. This emphasizes that chronic gastritis has a greater chance of causing onset of gastric cancer than the presence of *H. pylori* itself. Furthermore, genetic factors, toxicity of the pathogen, and environmental factors are intertwined in the development of *H. pylori*-induced gastric cancer from inflammation, thus alleviation or treatment of inflammation could form the basis of prevention of gastric cancer [28,31,32].

## 4. Cancer Prevention by Controlling *H. pylori* Associated Inflammation

Although debates still exist whether *H. pylori* infection is really class I carcinogen or not, *H. pylori* has been known to provoke and promote precancerous lesions like gastric adenoma and chronic atrophic gastritis with intestinal metaplasia as well as gastric cancer. Chronic persistent, uncontrolled gastric inflammations are possible basis for ensuing gastric carcinogenesis and *H. pylori* infection increased COX-2 expressions, which might be the one of the mechanisms leading to gastric cancer. Therefore, to know the implication of long-term treatment of anti-inflammatory drugs, rebamipide, a drug possessing radical scavenging action as well as prostaglandins induction, or nimesulide, a drug possessing selective COX-2 inhibition action, coxib, on *H. pylori*-associated gastric carcinogenesis, we infected C57BL/6 mice with *H. pylori*, especially after *N*-methyl-*N*-nitrosourea (MNU) administration to promote carcinogenesis and the effects of the long-term administration of rebamipide or nimesulide were evaluated. C57BL/6 mice were sacrificed 50 weeks after *H. pylori* infection. Colonization rates of *H. pylori*, degree of gastric inflammation and other pathological changes including atrophic gastritis and metaplasia, serum levels and mRNA transcripts of various mouse cytokines and chemokines, and NF-κB binding activities, and finally the presence of gastric adenocarcinoma were compared between *H. pylori* infected group (HP), and *H. pylori* infected group administered with long-term rebamipide containing pellet diets (HPR) or *H. pylori* infected group administered with long-term nimesulide containing pellet diets (HPN). As results, gastric mucosal expressions of ICAM-1, HCAM, MMPs, and transcriptional regulations of NF-κB binding were all significantly decreased in HPR and HPN group than in HP alone group. Multi-probe RNase protection assay showed the significantly decreased mRNA levels of apoptosis related genes and various cytokines genes like IFN-γ, RANTES, TNF-α, TNFR p75, IL-1β in HPR group. In the experiment designed to provoke gastric cancer through MNU treatment with *H. pylori* infection, the incidence of gastric carcinoma was not changed between HP and HPR group, but significantly decreased in HPN group, signifying that modulation of *H. pylori*-associated inflammation can be included in broad spectrum of the chemoprevention of *H. pylori*-associated gastric carcinogenesis based on the link between chronic inflammation and gastric carcinogenesis, leading to the conclusion that long-term administration of anti-inflammatory drugs should be considered in the treatment of *H. pylori* since they showed the clear molecular and biologic advantages with possible chemopreventive effect against *H. pylori*-associated gastric carcinogenesis. In our trial, though a specific selective COX-2 inhibitor, nimesulide was better than a nonspecific modulator, rebamipide, the consideration of long-term administration rendered rebamipide preferable than nimesulide under the warranty of long-term safety. If the final concrete proof showing the causal relationship between *H. pylori* infection and gastric carcinogenesis could be obtained, that will shed new light on chemoprevention of gastric cancer, that is, that gastric cancer could be prevented through either the eradication of *H. pylori* or lessening the inflammation provoked by *H. pylori* infection in high risk group [33-35]. Overproduction of nitric oxide via iNOS has been suggested to be a significant pathogenic factor in *H. pylori* induced gastritis, for which iNOS deficient mice (iNOS^-/-^) and wild-type littermates were challenged with a combination treatment comprising MNU administration and *H. pylori* infection. After 50 weeks, the incidence of gastric adenocarcinoma was significantly lower in iNOS^-/-^ compared with iNOS wild-type mice and iNOS and 3-nitrotyrosine expression was greater in tumor tissues than in non-tumour tissues. Conclusive findings that lowering of iNOS derived NO levels may be an important clinical strategy in the prevention of *H. pylori* associated gastric cancer opened the possibility of cancer prevention with antioxidants therapy [36].

Further studies were followed in our laboratory to show that appropriate intervention of suppressing or eliminating *H. pylori*-associated inflammation might be essential in cancer prevention. For instance, EGCG pretreatment effectively rescued gastric mucosal cells from the *H. pylori*-induced apoptotic cell death and DNA damage, and administration of this catechin enhanced gastric epithelial cell proliferation. This disturbance of *H. pylori*-induced host cell signaling by EGCG attenuated the synthesis of the pro-inflammatory mediator, hydroxyeicosatetraenoic acid [37]. Since ginseng, the root of *Panax ginseng* C.A. Meyer, has been reported to possess anti-adhesion or antimicrobial activity against *H. pylori*, the protective effect of red ginseng extracts against *H. pylori*-induced cytotoxicity and DNA damage was studied by our group [38]. Red ginseng extracts significantly attenuated both *H. pylori*-induced DNA damage assessed by comet assay and apoptosis measured by DNA fragmentation. Inactivation of ERK1/2 signaling and attenuation of caspase-3 activation and PARP cleavage were revealed with red ginseng extracts against *H. pylori* infection. Conclusively, red ginseng extracts showed significant gastroprotective effects against *H. pylori*-associated gastric mucosal cell damage, suggesting that red ginseng could be used as a medicinal phytonutrient against *H. pylori* infection.

## 5. Conclusions

**Figure 3.**
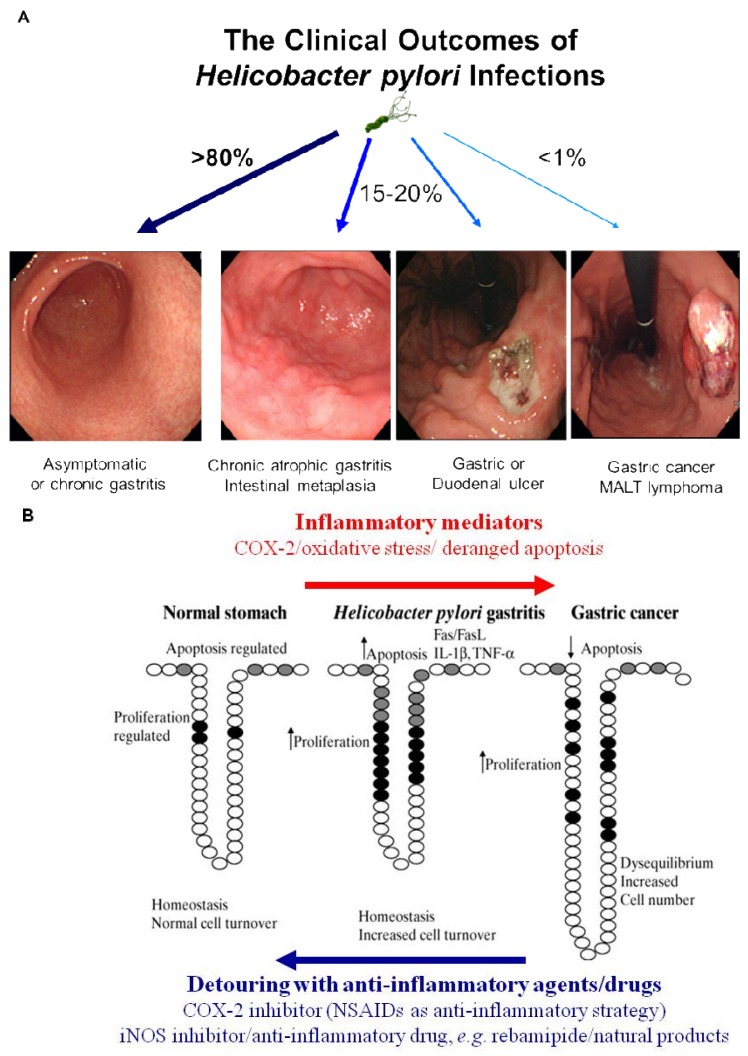
Detouring the road to *H. pylori*-associated carcinogenesis through alleviating gastric inflammation. (**A**) In spite of IARC definition that *H. pylori* infection is class I carcinogen, more than 80% among population infected with *H. pylori* remains asymptomatic, about which no clear explanation has not been forwarded. On the other hand, *H. pylori* infection is apparently associated with the emergence of precancerous lesion and some gastric malignancy. Host genetic polymorphisms, environmental factors, *H. pylori* strains, and host immune response might affect the outcome, but the common fact is that chronic gastric inflammation is the fundamental basis for *H. pylori*-associated gastric carcinogenesis. (**B**) Multiple lines of evidence had suggested the detouring the unpleasant journey to *H. pylori*-associated gastric carcinogenesis seems to be the effective strategy to prevent *H. pylori*-associated gastric cancer, for which COX-2 inhibitor, antioxidants, anti-inflammatory drug, phytoceuticals, and others had been documented.

Simple removal of *H. pylori* was proven to be insufficient for the prevention of gastric cancer in the current era of ambiguous guideline of *H. pylori* eradication, in spite of scattered publications showing a crosslink between *H. pylori* infection and gastric cancer and scant evidences of cancer prevention through the removal of the bug [4]. Therefore, nutritional intervention or potent anti-inflammatory agent can supply these limitations of gastric cancer prevention because strict control of *H. pylori*-associated gastric inflammation or oxidative stress shed the clear chance of cancer prevention. Rather than certain agents or drugs harboring risk of side effects with chronic administration, continuous intake of phytoceuticals or phytonutrients might provide higher chance of benefits through suppressing the activities of *H. pylori* [39]. However, while dietary habits or nutritional intake continue to rank as significant factors influencing the incidence of gastric cancer, there have been considerable scientific uncertainties about who will benefit, but who about will not benefit from nutrition, which might be due to inadequate knowledge about an individual's genetic background, the cumulative effect of nutrients on genetic expression profiles, ambiguous clinical differences between beneficiaries and non-beneficiaries and the lack of information about active protein induction [40]. Therefore, profuse and core knowledge about a nutrigenomic study to guide optimal dietary intervention and personal recommendations will provide an more essential basis for personalized dietary recommendations to prevent common multi-factorial diseases decades before their overt clinical manifestation. Conclusively, as seen in Figure 3A, *H. pylori* infection is apparently associated with the emergence of precancerous lesion and some gastric malignancy, though a small portion among infected population. Though host genetic polymorphisms, environmental factors, *H. pylori* strains, and host immune response might affect the outcome, but the common fact is that chronic gastric inflammation is the fundamental basis for *H. pylori*-associated gastric carcinogenesis. Therefore, the detouring the unpleasant journey to *H. pylori*-associated gastric carcinogenesis seems to be the effective strategy to prevent *H. pylori*-associated gastric cancer, for which optimal treatment strategy or agents should be developed more [40] (Figure 3B).
